# Genetic Characterisation of the *upp* Gene in *Bifidobacterium bifidum* PRL2010

**DOI:** 10.1111/1751-7915.70189

**Published:** 2025-07-06

**Authors:** Giulia Longhi, Silvia Petraro, Christian Milani, Chiara Tarracchini, Chiara Argentini, Laura Maria Vergna, Gabriele Andrea Lugli, Leonardo Mancabelli, Ciaran Lee, Francesca Turroni, Douwe van Sinderen, Marco Ventura

**Affiliations:** ^1^ Laboratory of Probiogenomics, Department of Chemistry, Life Sciences, and Environmental Sustainability University of Parma Parma Italy; ^2^ Microbiome Research Hub University of Parma Parma Italy; ^3^ Department of Medicine and Surgery University of Parma Parma Italy; ^4^ APC Microbiome Institute and School of Microbiology, Bioscience Institute National University of Ireland Cork Ireland

**Keywords:** *Bifidobacterium*, microbe‐microbe interaction, microbiota

## Abstract

Bifidobacteria are key members of the human gut, especially during infancy. The ability of bifidobacteria to outcompete other members of the microbial communities encountered in this highly competitive human gut environment represents a key example of their evolutionary and ecological success. In the current report, we investigated the highly conserved bifidobacterial *upp* gene, which encodes the uracil phosphoribosyltransferase and which is involved in the pyrimidine salvage pathway. Phylogenetic analysis incorporating 107 bifidobacterial *upp* sequences, representing all currently known *Bifidobacterium* taxa, indicates that this gene followed an evolutionary route that apparently deviates from that of the 16S rRNA gene. In addition, the *upp* gene may support bifidobacterial survival in environments with limited uracil availability, potentially providing a competitive advantage under nutrient‐restricted conditions.

## Graphical abstract

In this study, we examined the highly conserved *upp* gene in bifidobacteria, which encodes uracil phosphoribosyltransferase and plays a role in the pyrimidine salvage pathway. Specifically, we explore its evolutionary trajectory in bifidobacteria, shedding light on its role in microbial competition in the human gut.

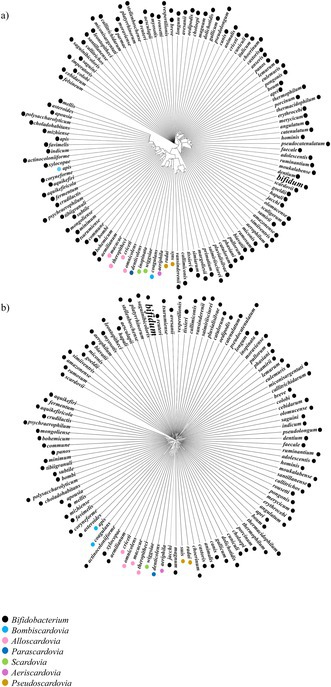

## 1 Introduction

Bifidobacteria are prevalent members of the microbial communities residing in the human gut, being particularly abundant during the first 6 to 12 months after birth until the weaning period (Milani et al. 2017; Alessandri et al. 2021). In this context their presence and associated metabolic activities are crucial for modulation of the host immune system and systemic metabolism (Milani et al. 2017; Turroni et al. 2021). The infant gut microbiota is represented by various assemblies of microbes, many of which are maternally inherited, and based on conserved compositional patterns several so‐called infant community state types, ICSTs, have been identified (Lugli et al. 2023). Such ICSTs include microbes engaged in positive trophic interactions through cross‐feeding activities, that is, where one microbe generates chemical compounds from the metabolism of environmental substrates that are then utilised by other members of these ICSTs as nutrients. However, in such an environment, enteric bacteria compete for the retrieval of nutrients that are essential for their anabolic reactions and possessing alternative pathways for such vital metabolic activities are expected to provide a selective advantage.

Nucleotide anabolism typically represents a housekeeping function of a bacterium, and it is of vital importance that such metabolic activities will not be hampered by a shortage of key pyrimidine and purine precursors. Notably, to mitigate such a risk many microbial taxa including bifidobacteria possess a pyrimidine salvage pathway encompassing the *upp* gene, encoding uracil phosphoribosyltransferase which catalyses the conversion of uracil and 5′‐phosphoribosyl‐alpha‐1′ pyrophosphate to uridine 5′‐monophosphate (UMP) and pyrophosphate (Villela et al. 2013). UMP is a common precursor of all pyrimidine bases and nucleosides (Warner et al. 2014), making the *upp* gene product a key enzyme in the pyrimidine salvage pathway by allowing direct reutilisation of uracil bases and ensuring energy and resource saving for the microbial cell (Villela et al. 2011).

Beyond its metabolic role, *upp* has been widely exploited as a genetic tool in bacteria for both counter‐selection and auxotrophy‐based strategies. In counter‐selection systems, *upp* enables sensitivity to toxic uracil analogs such as 5‐fluorouracil (5‐FU); strains expressing a functional *upp* gene convert 5‐FU into toxic metabolites, leading to cell death, while *upp* mutants are 5‐FU resistant. This feature has been used to select for successful allelic replacements or plasmid curing events (Rudland et al. 1989; Murphy and Saltikov 2007). Although we did not directly assess 5‐FU resistance in this study, *upp* can serve as a selectable marker in auxotrophic systems, where uracil supplementation allows growth of only those strains that possess an active salvage pathway. These characteristics make *upp* a versatile and widely used tool in microbial genetics, including applications in *E. coli* , *Enterococcus*, and certain probiotic strains such as *Bifidobacterium longum* and *Lactobacillus* species (Paliy et al. 2009; Frontzek et al. 2020).

Although *B. bifidum* PRL2010 likely possesses a complete de novo pyrimidine biosynthetic pathway, as supported by the presence of genes encoding enzymes such as dihydroorotate dehydrogenase, the relative contribution of de novo synthesis versus salvage remains unclear and may depend on environmental context (Bottacini et al. 2010; O'Connell Motherway et al. 2011). In nutrient‐rich conditions, de novo synthesis may suffice; however, in uracil‐limiting niches, the salvage pathway may become essential for efficient nucleotide recycling and competitive survival. This redundancy likely provides metabolic flexibility to adapt to dynamic gut environments. This study aimed to investigate the occurrence of the *upp* gene in genomes of enteric bifidobacteria and to provide a molecular characterisation of this genetic element as well as assessing its importance in enhancing the ecological fitness of bifidobacteria in the human gut.

## 2 Results and Discussion

### 2.1 Genomic Distribution of *upp*‐Encoding Genes in Bifidobacteria

*upp* homologues are widely conserved in the chromosome sequences of the *Bifidobacterium* genus. Amino acid alignment with deduced bifidobacterial Upp proteins showed the classical phosphoribosyl transferase (PRT) type I domain (InterPro entry IPR0008369) involved in pyrimidine metabolism. This domain is found in several phosphoribosyl‐transferase enzymes and proteins that regulate nucleotide synthesis and recovery pathways, including uracil phosphoribosyltransferase (EC 2.4.2.9) (Tables S1 and S2) (Islam et al. 2007). In line with this, the three‐dimensional structure of the Upp protein from *B. bifidum* PRL2010 was predicted using AlphaFold2 (via the ColabFold implementation), resulting in a high‐confidence model (pLDDT > 90) (Figure S2). Structural comparison of *upp*‐encoding gene with protein sequences clustered using the MMseqs2 algorithm revealed a significant fold‐level conservation with known uracil phosphoribosyltransferases from several other bacterial species (predominantly belonging to the *Salmonella* and *Escherichia* genera), suggesting a conserved functional evolution.

Moreover, analysis of 3285 bifidobacterial genomes, encompassing all 107 currently recognised species of the genus *Bifidobacterium* revealed that the *upp* gene is part of the core genome of this genus (aa similarity above 83%), and thus part of the bifidobacterial house‐keeping gene arsenal (Figure S1a, Tables S3 and S4). In addition, the *upp* gene was shown to be highly conserved in genomes of other members of the Bifidobacteriacea family, that is, *Scardovia*, *Parascardovia*, *Aeroscardovia*, *Alloscardovia*, *Bombiscardovia*, *Pseudoscardovia*, and *Metascardovia* genera as well as in other Actinomycetota members (Lugli et al. 2017). Furthermore, homology analysis revealed that the *upp* gene is also conserved across species belonging to different genera of intestinal bacteria, suggesting a potentially shared functional role among members of the gut microbiota (Table S5).

Notably, a phylogenetic analysis performed using these identified *upp* homologues revealed a different co‐phylogeny trend with another classical molecular marker, that is, the 16S rRNA gene, indicating that the bifidobacterial *upp* gene was horizontally acquired (Figure S1b).

### 2.2 Transcriptomic Analysis of the Bifidobacterial *upp* Gene

**FIGURE 1:**
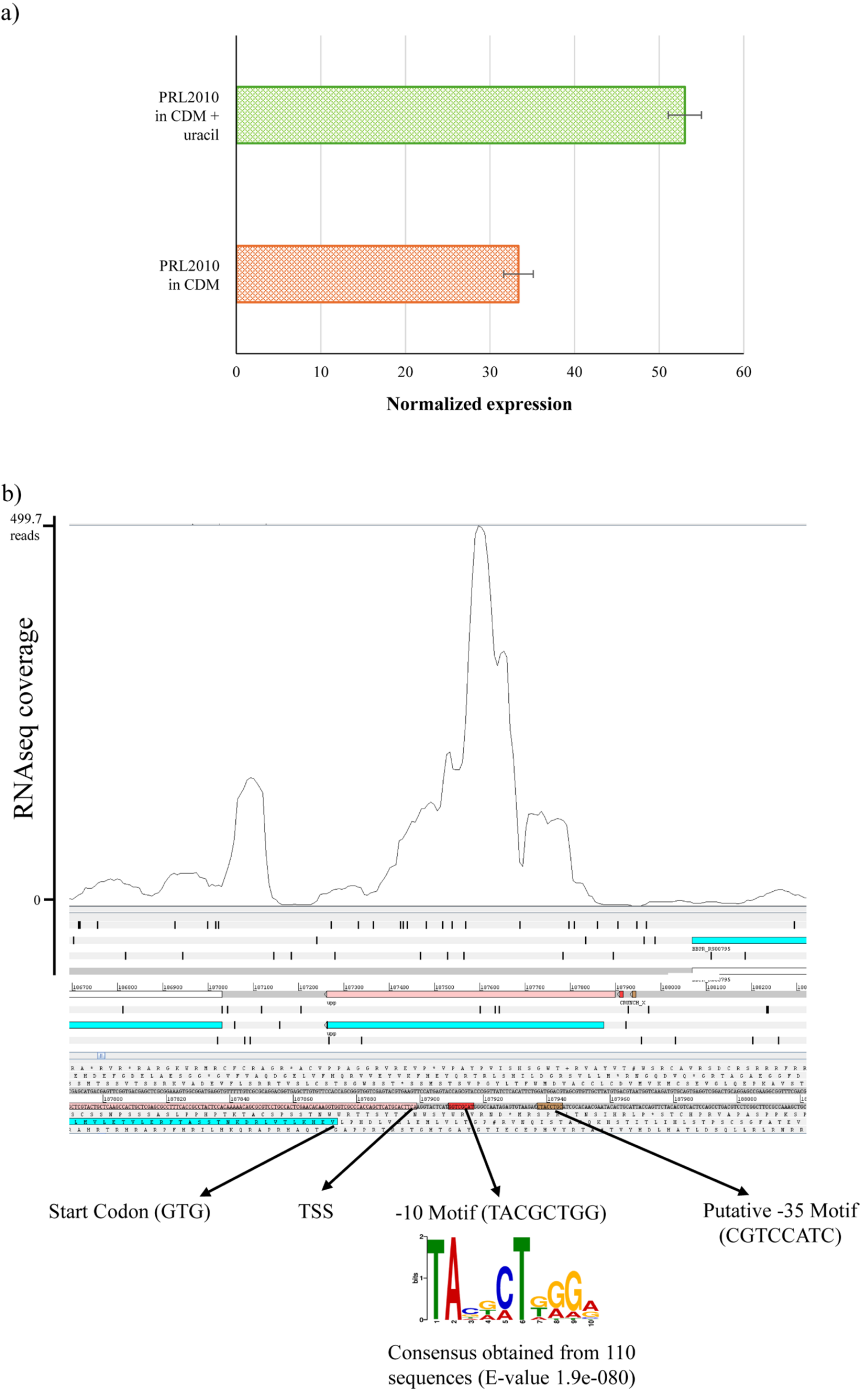
Transcriptomic analysis of the *upp* gene of *B. bifidum* PRL2010. The plot in panel (a) shows the transcription level of the *upp* gene under different conditions: with or without uracil supplementation. The histograms indicate the transcriptional levels of *upp* by qRT‐PCR for *B. bifidum* PRL2010 wild type. Statistically significant differences were analysed using the Kruskal–Wallis test. Panel (b) displays the identification of the *B. bifidum* PRL2010 *upp* transcription initiation site (TSS) using RNAseq data. The predicted ‐10 and ‐35 promoter regions are underlined, the TSS is marked, and the *upp* start codon (GTG) is indicated.

To investigate whether the expression of the bifidobacterial *upp* gene is influenced by specific growth conditions, we focused on *Bifidobacterium bifidum* PRL2010, a well‐characterised prototype species/strain of the infant gut microbiota (Turroni et al. 2019; Fontana et al. 2022), which has been extensively used as a model organism to study host–microbe interactions and gene function. We analysed the level of *upp* gene expression in PRL2010 wild‐type by qPCR when this strain was grown in a chemically defined medium (CDM) with or without the addition of uracil (Figure 1a). When this growth medium contained uracil, *upp* gene transcription was shown to be increased just 1.6‐fold compared to the reference condition (uracil being absent), although this observed difference is not deemed to be a significant transcriptional change for prokaryotes (Harshitha and Arunraj 2021). Therefore, it appears that *upp* gene is not under uracil‐dependent transcriptional control but rather represents a constitutively transcribed gene. Nonetheless, while *upp* expression seems unaffected by extracellular uracil, it remains plausible that uracil uptake in PRL2010 wild type is facilitated by membrane transporters whose expression or activity may be influenced by uracil availability. To date, however, no dedicated uracil transporter has been experimentally identified in *B. bifidum* , and further studies are required to clarify the molecular mechanisms involved in uracil import.

**FIGURE 2:**
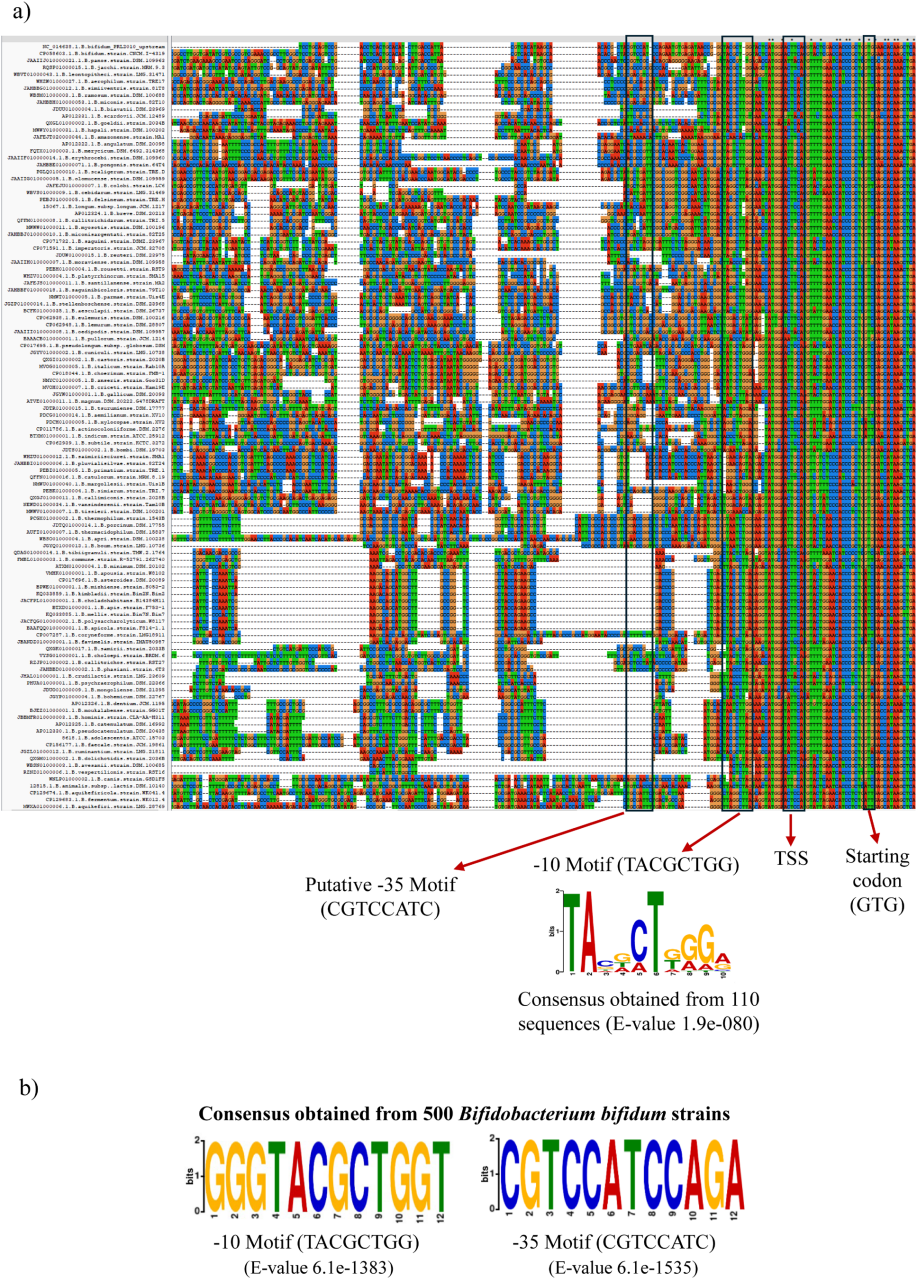
Comparative analysis of the *upp* promoter region across members of the *Bifidobacterium* genus. Panel (a) shows a multiple sequence alignment of the upstream regions of the *upp* gene from 110 bifidobacterial species. Conserved motifs and promoter elements are framed. Panel (b) represents sequence similarity of the *upp* upstream region in *B. bifidum* PRL2010 compared with 500 other *B. bifidum* strains.

To determine the transcriptional start site (TSS) of the *upp* gene, RNAseq experiments were performed with RNA extracted from *B. bifidum* PRL2010 cells cultivated on CDM. The generated data allowed us to predict the 5′ end of the *upp*‐encompassing transcript, that is, the transcriptional start site or TSS, by inspecting the RNAseq alignment coverage, as described previously (Bottacini et al. 2017) (Figure 1b). Although a canonical Shine‐Dalgarno (SD) sequence (e.g., AGGAGG) is not present, a non‐canonical motif (ACCACC) is placed upstream of the predicted start codon (GTG). This sequence likely serves as a functional SD sequence, as reported for other *Bifidobacterium* species, where ribosome binding sites often diverge from the classical consensus but still support efficient translation initiation (Lee and O'Sullivan 2010; Milani et al. 2017) (Figure 1b). Additionally, to identify ‐10 and ‐35 promoter motifs, we aligned the DNA sequences upstream of the identified TSS of the *upp* gene of PRL2010 with corresponding *upp* upstream sequences of other 110 bifidobacterial species, representing all the taxa of the *Bifidobacterium* genus (Turroni et al. 2016; Alessandri et al. 2021) (Figure 2a). These sequences were used as input for the MEME's motif analysis (https://meme‐suite.org/meme) and additionally confirmed using ProPr promoter prediction tool (http://propr.molgenrug.nl/) (Bailey et al. 2015). As reported in Figure 2a, we identified ‐10 and putative ‐35 promoter consensus sequences consistent with what described previously for other bifidobacterial genes (Bottacini et al. 2017). Further in silico analyses of the *upp* gene revealed that the gene was flanked at its 3′ end by an inverted repeat that may function as a rho‐independent transcriptional terminator. Altogether such findings clearly indicate that the *upp* gene possesses its own promoter region. Notably, comparison of the upstream region of *upp* gene of PRL2010 with those of other 500 *B. bifidum* genomes revealed a high level of DNA homology, suggesting that the *upp* promoter region is highly conserved in this taxon (Figure 2b).

### 2.3 Evaluation of the Function of the *upp* Gene of *B. bifidum* PRL2010

**FIGURE 3:**
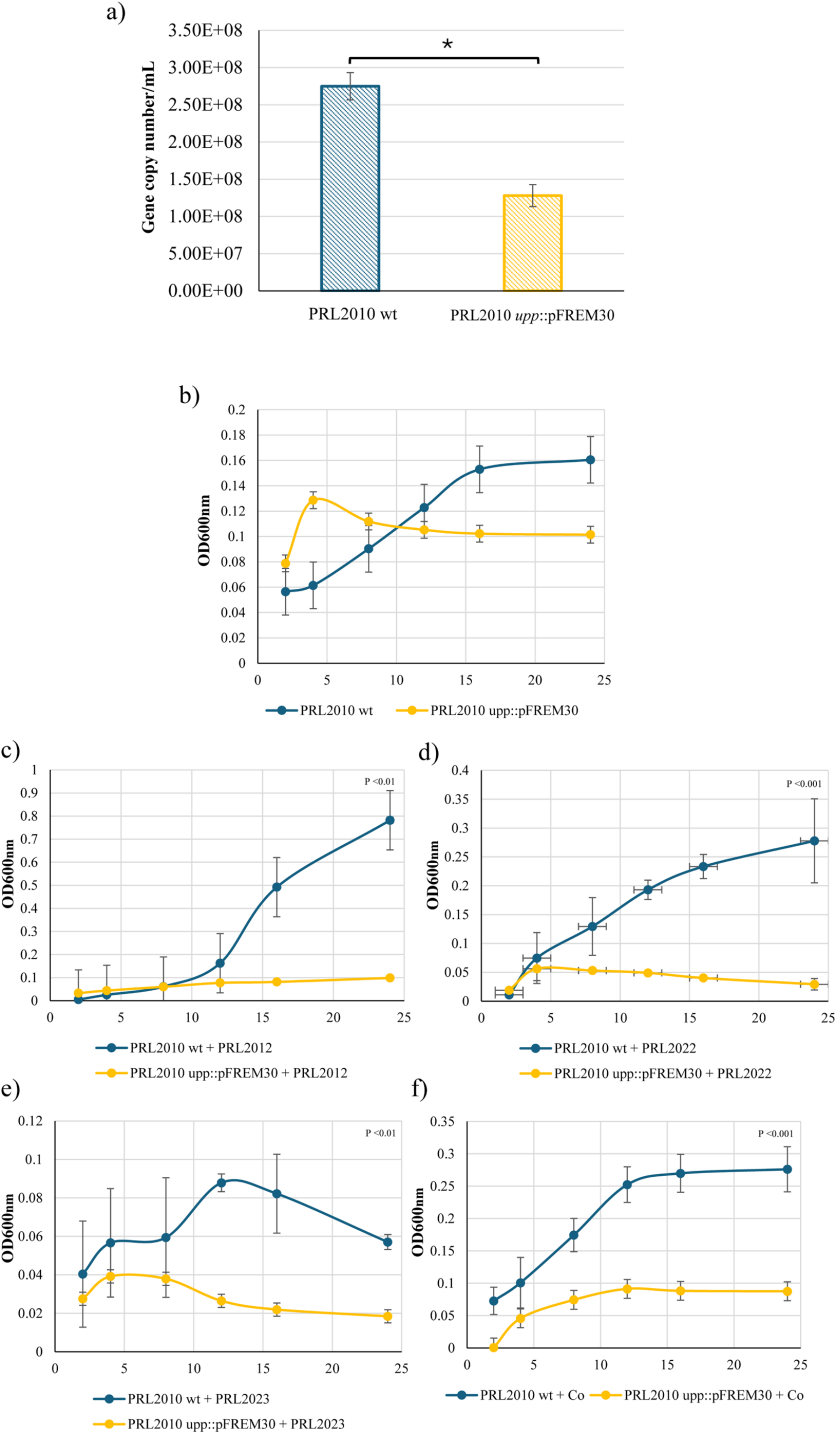
Impact of *upp* gene disruption on growth performance of *B. bifidum* PRL2010. Panel (a) shows the growth performance of *B. bifidum* PRL2010 wild type versus the mutant strain when cultivated in chemically defined medium (CDM) without uracil, as assessed by qPCR. Panels (b–f) display the time‐course growth curves of PRL2010 wild type and the mutant strain cultured individually or in association with other bifidobacterial species: *Bifidobacterium breve* PRL2012 (panel c), *Bifidobacterium longum* PRL2022 (panel d), *Bifidobacterium adolescentis* PRL2023 (panel e) and in combination with all three species (indicated with ‘Co’) (panel f) as measured by optical density at 600 nm (OD_600nm_). *p < 0.05.

To corroborate the in silico data pertaining to the predicted biological function exerted by the *upp* gene, we generated an *upp* insertion mutant in *B. bifidum* PRL2010, designated as *B. bifidum* PRL2010 *upp*::pFREM30, following a previously described protocol (Rizzo et al. 2024). Briefly, the mutant was generated by introducing the non‐replicative plasmid pFREM30, carrying a chloramphenicol resistance cassette, into *B. bifidum* PRL2010 via electroporation, and selecting for successful recombinants. To disrupt the *upp* gene and generate a stable insertional mutant, we selected a target site within the 5′ region of the coding sequence, as this increases the likelihood of complete gene inactivation by preventing the synthesis of a functional protein. The insertion site was chosen to avoid overlaps with regulatory elements or adjacent genes, in order to minimise potential polar effects on neighbouring loci. Furthermore, the selected region was unique within the genome to ensure site‐specific insertion and to prevent off‐target effects. To assess the effect of the insertional mutation, we evaluated potential phenotype changes in the mutant strain compared to the wild type. In this context, a significant reduction in the growth of the mutant compared to the wild type was observed when cultivated in CDM without uracil, as assessed by qPCR (Kruskal–Wallis test *p* < 0.05) (Figure 3a). This reduced growth performance is likely due to the activity of the de novo synthesis pathway, a more complex and energy‐intensive alternative. This pathway forms the pyrimidine from basic precursors, converting it into UMP, which then provides the building blocks for nucleic acid synthesis. Notably, the growth performance of the wild type strain remained similar regardless of the presence or absence of exogenous uracil (2.75 × 10^8^ gene copy number/mL vs. 1.71 × 10^8^ gene copy number/mL, respectively), suggesting that *B. bifidum* PRL2010 can efficiently rely on endogenous uracil recycling, and that UPRTase activity does not critically depend on extracellular uracil availability under these conditions.

Furthermore, co‐cultivation experiments of PRL2010 wild type and its isogenic *upp* mutant were performed with other bifidobacterial strains commonly found as colonisers of the human gut considered prototype strains, that is, *Bifidobacterium breve* PRL2012, *Bifidobacterium longum* PRL2022, and *Bifidobacterium adolescentis* PRL2023 (Alessandri et al. 2023; Argentini et al. 2024a, 2024b). These experiments were conducted using the Cerillo Co‐Culture Duet System, which allows metabolite exchange between microorganisms without direct contact. Both the PRL2010 wild type and the *upp*::pFREM30 mutant were cultivated in CDM alone and in bi‐ or co‐associations with the same bifidobacterial species previously tested. Bacterial growth was monitored by measuring OD_600nm_ at different timepoints. Under these conditions, the mutant exhibited a shorter lag phase, followed by accelerated exponential growth during the first 4 h of incubation, before slightly decreasing and reaching a stationary phase (Figure 3b). In contrast, PRL2010 wild type showed a more balanced growth pattern, with a well‐defined exponential phase that exceeds that of the mutant in terms of final OD_600nm_ reached. Although both strains rely on the de novo pathway for pyrimidine biosynthesis in the absence of uracil, the wild type may benefit from tighter regulatory control of nucleotide metabolism and a more optimal intracellular balance of nucleotide pools. Conversely, the *upp* mutant might experience subtle metabolic stress or altered feedback regulation, which could explain the transient early acceleration followed by a lower overall growth performance. In addition, when the PRL2010 *upp*::pFREM30 mutant was co‐cultivated with *B. breve* PRL2012 and *B. longum* PRL2022, it exhibited a drastic reduction in growth, which was statistically significant compared to the wild type (Mann–Whitney *U* test *p* < 0.01 and *p* < 0.001, respectively) (Figure 3b). These observations suggest that these bifidobacterial partners may release metabolites that promote the growth of the wild type strain, while the *upp* mutant is unable to benefit from such compounds. Moreover, the reduction may be attributed to increased metabolic competition in the absence of exogenous uracil. In this context, the presence of an intact salvage pathway in the wild type strain may enhance intracellular nucleotide recycling efficiency, thereby strengthening its capacity to cope with nutrient stress. In contrast, the mutant strain, which lacks UPRTase activity, may be less metabolically adaptable, resulting in impaired growth during co‐cultivation with other bifidobacteria. Notably, when co‐cultivated with *B. adolescentis* PRL2023, the wild type strain displayed a less stable growth pattern compared to other co‐cultures, possibly due to stronger competition for limited nutrients in the minimal medium, likely reflecting the more competitive metabolic profile of *B. adolescentis* . Similarly, when co‐associated with all bifidobacterial species, the *upp* mutant showed a significantly reduced growth performance compared to the PRL2010 wild type (Mann–Whitney *U* test *p* < 0.001) (Figure 3b). Such data clearly imply that the *upp* gene of PRL2010 is involved in the uracil salvage pathway. Furthermore, our data indicate that this gene plays an important role in facilitating colonisation and establishment of bifidobacterial taxa in uracil deprived environments or in allowing bifidobacterial growth in complex and highly competitive microbial communities, such as the human gut.

## 3 Conclusions

Bifidobacteria are key microbial members of the infant gut microbiota and in this environment, they may engage in molecular interactions with other microbes for the utilisation of nutrients. In this context, in a very competitive environment such as the human gut, engraftment and subsequently successful long‐term colonisation of bacteria are also based on their prototrophic features for the biosynthesis of macromolecules such as nucleic acids. Notably, the fact that the *upp* gene is part of the core structure of bifidobacterial genomes further reinforces the notion that it performs a house‐keeping function for bifidobacteria, possibly linked to their ecological adaptation within complex microbial communities. In this context, the occurrence of a salvage pathway for the recycling of pyrimidines represents a crucial metabolic feature of bifidobacteria, enhancing their ecological fitness and thus aiding their stability in the human gut. Although direct quantification of uracil levels in the intestinal environment is still limited, previous studies suggest that free uracil may be present in the gut because of host cell turnover and microbial metabolic activity. For instance, some members of the gut microbiota are known to secrete pyrimidine bases or nucleosides as metabolic by‐products (Lee et al. 2013). Furthermore, studies on gut microbial metabolism have indicated that nucleotides and their derivatives, including uracil, may accumulate in the gut environment depending on diet, microbial activity, and host‐microbiota interactions (Donia and Fischbach 2015; Louis and Flint 2017). This provides an accessible pool of pyrimidines that can be exploited by uracil‐scavenging bacteria, such as bifidobacteria, thereby offering a potential selective advantage in nutrient‐limited or competitive conditions. In addition, such genetic elements further corroborate the strict ecological adaptation of bifidobacteria to the mammalian gut where the bifidobacterial *upp* gene may contribute to the ability of these gut commensals to effectively compete for nucleic acid constituents within a very complex microbial community. Since complementation of the mutant strain would allow a more detailed investigation of the *upp* gene function, this approach is not currently feasible and will be explored in further studies.

## 4 Materials and Methods

### 4.1 Genomic Analysis

Genomes belonging to the *Bifidobacterium* genera were analysed in this study. Complete and/or draft genomes were downloaded from the NCBI Genome database, using taxonomic membership updated at the time of analysis as selection criteria. An analysis was then performed with the Basic Local Alignment Search Tool (BLASTp) of the proteins (cutoff value e‐ of 1 × 10^−10^) against the *upp* sequence of the reference genome for each species. The amino acid sequences of Upp proteins, obtained from the reference genomes of each species considered, were analysed in their protein domains using InterProScan 5.68–100.0 (Quevillon et al. 2005).

The *upp* and 16S rRNA gene sequences of the type strains of each of the species belonging to the genus *Bifidobacterium*, as well as the genera *Bombiscardovia*, *Alloscardovia*, *Parascardovia*, *Scardovia*, *Aeriscardovia* and *Pseudoscardovia*, were aligned using Clustal Omega (v1.2.4) (Sievers and Higgins 2014). The alignments were then used to construct preliminary phylogenetic trees using the Neighbour‐Joining method, as implemented in Clustal Omega, and subsequently visualised using the Interactive Tree Of Life v5 (iTOL) platform (Letunic and Bork 2021).

### 4.2 Cultivation of *B. bifidum* PRL2010 Wild Type in a CDM

*Bifidobacterium bifidum* PRL2010 was cultivated in a modified de Man–Rogosa–Sharpe (MRS) medium without glucose yet supplemented with 0.05% L‐cysteine hydrochloride and 2% lactose (mMRS) in an anaerobic chamber (concept 400, Ruskinn) at 37°C. After overnight culture, the wild type was inoculated in a previously formulated medium containing (per litre of distilled water) 4.0 g of sodium acetate; 1.0 g of tri‐ammonium citrate; 2.0 g of KH_2_PO_4_; 2.0 g of K_2_HPO_4_; 0.5 g of MgSO_4_.7H_2_O; 0.05 g of MnSO_4_.H_2_O; 0.02 g of FeSO_4_.7H_2_O; 0.2 g of CaCl_2_; 20 mg of adenine; 40 mg of xanthine; 0.4 g of cysteine; 0.3 g of aspartic acid; 0.3 g of glutamic acid; 0.2 g of each of the following amino acids: alanine, arginine, glycine, histidine, isoleucine, leucine, lysine, methionine, phenylalanine, proline, serine, threonine, tryptophan, tyrosine, and valine; 0.5 g of orotic acid; 0.5 mg of p‐aminobenzoic acid; 0.5 mg of folic acid; 2.0 mg of nicotinic acid; 2.0 mg of Ca‐pantothenate; 1.0 mg of biotin; 2.0 mg of pyridoxal; 2.0 mg of riboflavin; and 1.0 mg of vitamin B12. Uracil is added to the medium to a final concentration of 0.04 mg/mL from a 0.02 mg/mL stock solution, based on preliminary optimisation to support the growth of the *upp* mutant strain and to provide sufficient pyrimidine supplementation without causing inhibitory effects. The medium was sterilised by filtration (0.22 μm).

### 4.3 Prokaryotic RNA Extraction and Sequencing

Total RNA was isolated as previously described (Turroni et al. 2016). Bacterial cell pellets were resuspended in 1 mL of QIAZOL (Qiagen, UK) and placed in a tube containing 0.8 g of glass beads (diameter, 106 μm; Sigma). Cells were lysed by alternating 2 min of stirring the mix on a bead beater with 2 min of static cooling on ice. The mixture was then centrifuged at 12,000 rpm for 15 min, and the RNA‐containing sample was retrieved from the upper phase. The RNA‐containing sample was further treated using a RNeasy minikit (Qiagen, Germany) according to the manufacturer's guidelines. The quality of the RNA was checked by using a Tape station 2200 (Agilent Technologies, USA). A spectrophotometer (Eppendorf, Germany) was employed to evaluate the RNA amount and purity. For RNAseq, from 100 ng to 1 μg of extracted RNA was treated to eliminate rRNA by using QIAseq FastSelect—5S/16S/23S according to the manufacturer's instructions (Qiagen, Germany). RNA yield upon rRNA depletion was checked using a Tape station 2200 (Agilent Technologies, USA). Afterward, a whole transcriptome library was made using the TruSeq Standard mRNA preparation kit (Illumina, San Diego, CA). Samples were loaded into a NextSeq high output v2.5 kit (150 cycles, single end) (Illumina) according to the protocol given. The obtained reads were filtered to remove low‐quality reads (minimum mean quality, 20; minimum length, 150 bp), as well as any residual ribosomal locus‐encompassing reads using the METAnnotatorX2 (Milani et al. 2021), yielding an average of 2,667,250 high‐quality reads (Table S2). Obtained reads were then aligned to the reference genome of each bifidobacterial strain used, using Bowtie2 software (Langdon 2015). Htseq‐counts script of HTSeq software in ‘union’ mode was used for the quantification of reads mapped to individual transcripts (Anders et al. 2015). Raw counts were then normalised using cpm (mapped reads) for filtering genes with low counts (cpm < 1) and trimmed mean of M values (TMM) for statistically robust differential gene expression analysis through the EdgeR package (Robinson et al. 2010). Evaluation of expression changes was calculated for each gene as log2 fold change (logFC) of average expression between the control and ‘treated’ samples. Moreover, a Volcano plot was created to simultaneously visualise expression changes (log fold change) and their statistical significance (p‐value) for each comparison.

### 4.4 qPCR‐Based Analysis of *upp* Gene Expression

Part of the obtained bacterial RNA was utilised for *upp* gene expression analysis by quantitative real‐time PCR (qPCR). Specifically, 500 ng of total RNA from each sample was reverse transcribed to cDNA using the iScript Select cDNA Synthesis Kit (Bio‐Rad Laboratories) with the following thermal cycle: 5 min at 25°C, 30 min at 42°C, and 5 min at 85°C. At the end of the process, aliquots of 25 ng/μL cDNA were amplified in a total volume of 10 μL using the PowerUp SYBR Green Master mix (Thermo Fisher Scientific), along with the forward and reverse primers (5 pmol each). Real‐time PCR was performed using the CFX96 system (Bio‐Rad, CA, USA). Fluorescence was monitored at the end of each extension step. Melting curve analysis was performed at the end of each amplification cycle. Data analysis was performed using the relative standard curve method (Bustin et al. 2005). The expression data are reported as the ratio between each investigated mRNA and mRNA belonging to three bifidobacterial housekeeping genes.

### 4.5 Transcriptome Analysis of *B. bifidum* PRL2010 Cultivated in CDM

*B. bifidum* PRL2010 wild type was cultivated as previously described in CDM supplemented with 2% lactulose without uracil to reach a final Optical Density at 600 nm (OD_600nm_) of 0.1 and incubated at 37°C for 6 h under anaerobic conditions (2.99% H_2_, 17.01% CO_2_ and 80% N_2_). After incubation, cells were harvested by centrifugation at 5000 × *g* for 5 min when the mid‐exponential growth phase was reached, and they were subsequently subjected to RNA extraction and sequencing. The experiments were carried out in triplicate.

### 4.6 Construction of an *B. bifidum* PRL2010 Insertion Mutant in the *upp* Gene

The general procedures used for DNA manipulation were those described in a previous study in which the protocol to transform *B. bifidum* PRL2010 was developed and optimised (Rizzo et al. 2024). Briefly, plasmid pFREM30 was obtained by amplifying the chloramphenicol gene (CmR) with primers CLMC_207 (5′–GATCGCTCTTCGGGGATTATAAAAGCCAGTCATTAGGCCTATCTGAC–3′) and CLMC_208 (5′–GATCGCTCTTCACTTATGAACTTTAATAAAATTGATTTAGACAATTGGAAGAG –3′), starting from the backbone of plasmid pFREM28 (Hoedt et al. 2021) with primers CLMC_009 (5′–GATCGCTCTTCTCCCCACCAAAACCGAAATCCAC–3′) and CLMC_010 (5′–GATCGCTCTTCTAAGGTGTGCTCCTTTCCCTCAC–3′). After purifying the DNA fragments, plasmid pFREM30 was assembled using Golden Gate Cloning with the Type IIS enzyme SapI. The resulting construct was introduced into *E. coli* EC101, and transformants were selected on LB plates containing 25 μg/mL chloramphenicol. Colonies were screened by colony PCR, and the correct plasmid was confirmed by sequencing. For the construction of plasmid pFREM30‐*upp*, the to‐be‐targeted internal region of the gene (locus tag BBPR_RS00790, from base 149 to base 413 of the *upp* gene) was amplified by PCR from chromosomal *B. bifidum* PRL2010 DNA employing the Q5 polymerase and primers LMV_1 (5′–gatcgagtgcacCATCATCGACAAGCCCATCGAAACC–3′) and LMV_2 (5′–gagatcctcgagTGGCGAGCATGGGGTCGAT–3′). Chromosomal DNA was extracted from *B. bifidum* PRL2010 cells using the GenElute Bacterial Genomic DNA kit (Merk, Germany) following the manufacturer's instructions. Plasmid DNA was isolated from *E. coli* EC101 using the GeneJET Plasmid Maxiprep Kit (Thermo Fisher Scientific, USA). The amplicon and plasmid were digested with XhoI and ApaLI, ligated by using the T4 DNA ligase, and introduced into *E. coli* EC101, as previously reported (Hanahan et al. 1991). To select for transformants, the manipulated cells were plated on LB supplemented with 25 μg/mL chloramphenicol, and the colonies were screened for the presence of the expected plasmid construct by colony PCR. Subsequently, an overnight culture of PRL2010 was inoculated into fresh mMRS broth supplemented with (7% v/w) sucrose and cultivated at 37°C until exponential growth phase (OD_600_ 0.5–0.6). Cells were collected by centrifugation at 4500 g for 10 min at 4°C, washed twice with a cold citrate‐sucrose buffer (0.5 M sucrose, pH 5.8), and then resuspended in 250 μL of the same buffer for electroporation. All these steps above were carried out while keeping the cells cold on ice. 100 μL of PRL2010 competent cells resuspended in citrate‐sucrose buffer was mixed with 1.5 μg of the plasmid in a precooled disposable electroporation cuvette with an interelectrode distance of 0.2 cm (Cell Project, Kent, UK). A resistance of 200 Ω, a capacitance of 25 μF, and a voltage of 2.5 kV were applied using a Gene Pulser apparatus (BioRad, UK). After electroporation, the bacteria were resuspended in 950 μL of sMRS and incubated for 3 h at 37°C in an anaerobic cabinet. Following this, the cells were plated on sMRS agar supplemented with 5 μg/mL chloramphenicol and incubated anaerobically at 37°C for 48 h. Potential mutants were screened by colony PCR using primers UPP_fw (5′–ACGTACTCGACCACCCGC–3′) and UPP_rv (5′–CATCGACGGCGCAGACGA–3′), which bind to the chromosomal gene outside the target region, and CLMC_022 (5′–GCCAACGTTTTCGCCAACG–3′), which binds to the (integrated) pFREM30 plasmid. The expected amplicon sizes were approximately 2605 and 517 bp, respectively. The amplicons were then sent for Sanger sequencing to further confirm that the integration event had occurred in the correct chromosomal position.

### 4.7 Quantitative Real Time PCR for Bacterial Cell Enumerations

*B. bifidum* PRL2010 wild type and *B. bifidum* PRL2010 *upp*::pFREM30 strains were cultivated first in mMRS and inoculated in CDM as previously described, with and without the supplementation of uracil for 72 h at 37°C in anaerobic conditions. Subsequently, cells from 6 mL of the culture were harvested by centrifugation at 6000 rpm for 8 min, and the obtained cell pellets were used for DNA extraction using the GenElute bacterial genomic DNA kit (Sigma‐Aldrich) following the manufacturer's guide. Bacterial enumerations were determined by qPCR based on a specific primer pair targeting a gene present in a single copy within the genome of *B. bifidum* PRL2010 (Bbif_0282Fw (5′–GCGAACAATGATGGCACCTA–3′) and Bbif_0282Rv (5′–GTCGAACACCACGACGATGT–3′)).

### 4.8 Impact of *upp* Gene Disruption on the Growth Performance of *B. bifidum* PRL2010

To evaluate whether the presence of metabolites released by other bifidobacteria can be exploited by the mutant strain to restore the metabolic/physiological functions potentially affected by the disruption of the *upp* gene, *B. bifidum* PRL2010 wild type and *B. bifidum* PRL2010 upp::pFREM30 strains were exposed to all the metabolites produced by other bifidobacterial strains, that is, *Bifidobacterium breve* PRL2012, *Bifidobacterium longum* PRL2022, *Bifidobacterium adolescentis* PRL2023, and a co‐association of these bifidobacterial strains. For this purpose, the Cerillo Co‐Culture Duet System (Cerillo, USA) was employed. This system allows two bacterial strains to be physically separated by a porous membrane (pore size of 0.2 μm), while still permitting the exchange of fluids, including metabolites, between the two cultivated strains. In detail, the bifidobacterial strains were independently grown overnight in nMRS supplemented with 2% lactose at 37°C under anaerobic conditions. Subsequently, the cells were enumerated using a Thoma cell counting chamber (Herka), diluted if necessary to reach an initial OD of 0.2, washed in PBS, and resuspended in fresh CDM supplemented with 2% lactulose. Then, 800 μL of each bifidobacterial strain was aliquoted into the Cerillo Co‐Culture Duet System to establish a fluidic contact between *B. bifidum* PRL2010 *upp*::pFREM30 or the WT strain and each bifidobacterial strain. Cells were incubated at 37°C under anaerobic conditions for 24 h. Cell growth was evaluated by monitoring the optical density at 600 nm in continuous mode, with absorbance readings performed at 2‐h intervals over 24 h of growth, and each reading was preceded by 30 s of continuous shaking at medium speed. Cultures were grown in triplicates, and the resulting growth data were expressed as the average of these replicates.

### 4.9 *In Silico* Tertiary Structure Prediction of *upp* Gene

The three‐dimensional structure of the Upp protein from *B. bifidum* PRL2010 was predicted using AlphaFold2. The amino acid sequence was submitted in FASTA format, and a multiple sequence alignment was generated using MMseqs2. The top‐ranked model was selected based on the predicted Local Distance Difference Test (pLDDT) confidence scores.

### 4.10 Statistical Analyses

Differences in bacterial abundance in qPCR experiments, *upp* gene expression in qPCR‐based expression analyses, and growth assays in Cerillo Co‐Culture Duet System were evaluated by nonparametric independent‐samples Kruskal–Wallis test analysis using IBM SPSS Statistics for Windows.

## Author Contributions

**Giulia Longhi:** investigation, methodology, validation, writing – original draft, writing – review and editing. **Silvia Petraro:** data curation, writing – original draft, writing – review and editing. **Christian Milani:** conceptualization, formal analysis, data curation, writing – original draft. **Chiara Tarracchini:** data curation, formal analysis, software. **Chiara Argentini:** investigation. **Laura Maria Vergna:** investigation. **Gabriele Andrea Lugli:** writing – original draft, formal analysis, data curation, software. **Leonardo Mancabelli:** formal analysis, data curation, software. **Ciaran Lee:** investigation, methodology, validation. **Francesca Turroni:** conceptualization, methodology, supervision, writing – original draft, resources. **Douwe van Sinderen:** conceptualization, methodology, writing – original draft, writing – review and editing, supervision. **Marco Ventura:** conceptualization, methodology, supervision, funding acquisition, writing – original draft, writing – review and editing, resources.

## Conflicts of Interest

The authors declare no conflicts of interest.

## Supporting information


**Figure S1.** Phylogenetic analysis based on the *upp* and 16S rRNA gene sequences. Panel (a) represents the phylogenetic tree based on sequences of the *upp* gene from type strains belonging to the genus *Bifidobacterium* and other representative members of the Bifidobacteriaceae family. Panel (b) shows the corresponding phylogenetic tree constructed from the 16S rRNA gene sequences of the same strains.
**Figure S2.** Structural prediction and model confidence assessment of the Upp protein from *Bifidobacterium bifidum* PRL2010. Panel (a) shows the predicted three‐dimensional structure generated with AlphaFold2. Panel (b) depicts the Predicted Aligned Error (PAE) plot indicating the expected positional error (in Ångströms, Å) between residues *x* and *y* when the predicted and true structures are aligned on residue *y*. The colour gradient ranges from dark green (low error, high confidence) to light green (high error, low confidence).

**Table S1.** Analysis of protein domains in amino acid sequences of the genus *Bifidobacterium* encoding uracil phosphoribosyltransferase.
**Table S2.** Conserved domain identification in Upp proteins from *Bifidobacterium* species.
**Table S3.** Genomes of strains of the genus *Bifidobacterium* deposited on RefSeq and used in Blastp analyses.
**Table S4.** Blastp analysis output.
**Table S5.** Identification of Upp homologs in diverse gut‐associated bacterial taxa.

## Data Availability

The data that support the findings of this study are openly available in SRA database at https://www.ncbi.nlm.nih.gov/sra, reference number PRJNA1235520.
