# Supplementary material for: Genetic Characterisation of the upp Gene in Bifidobacterium bifidum PRL2010
Source: Microb Biotechnol. 2025 Jul 6;18(7):e70189. doi: 10.1111/1751-7915.70189 (PMC12230199; doi:10.1111/1751-7915.70189)
Supplement: Supplementary file 1 — Figure S1. Phylogenetic analysis based on the upp and 16S rRNA gene sequences. Panel (a) represents the phylogenetic tree based on sequences of the upp gene from type strains belonging to the genus Bifidobacterium and other representative members of the Bifidobacteriaceae family. Panel (b) shows the corresponding phylogenetic tree constructed from the 16S rRNA gene sequences of the same strains. Figure S2. Structural prediction and model confidence assessment of the Upp protein from Bifidobacterium bifidum PRL2010. Panel (a) shows the predicted three‐dimensional structure generated with AlphaFold2. Panel (b) depicts the Predicted Aligned Error (PAE) plot indicating the expected positional error (in Ångströms, Å) between residues x and y when the predicted and true structures are aligned on residue y. The colour gradient ranges from dark green (low error, high confidence) to light green (high error, low confidence). [file MBT2-18-e70189-s001.pptx]

## Slide 1
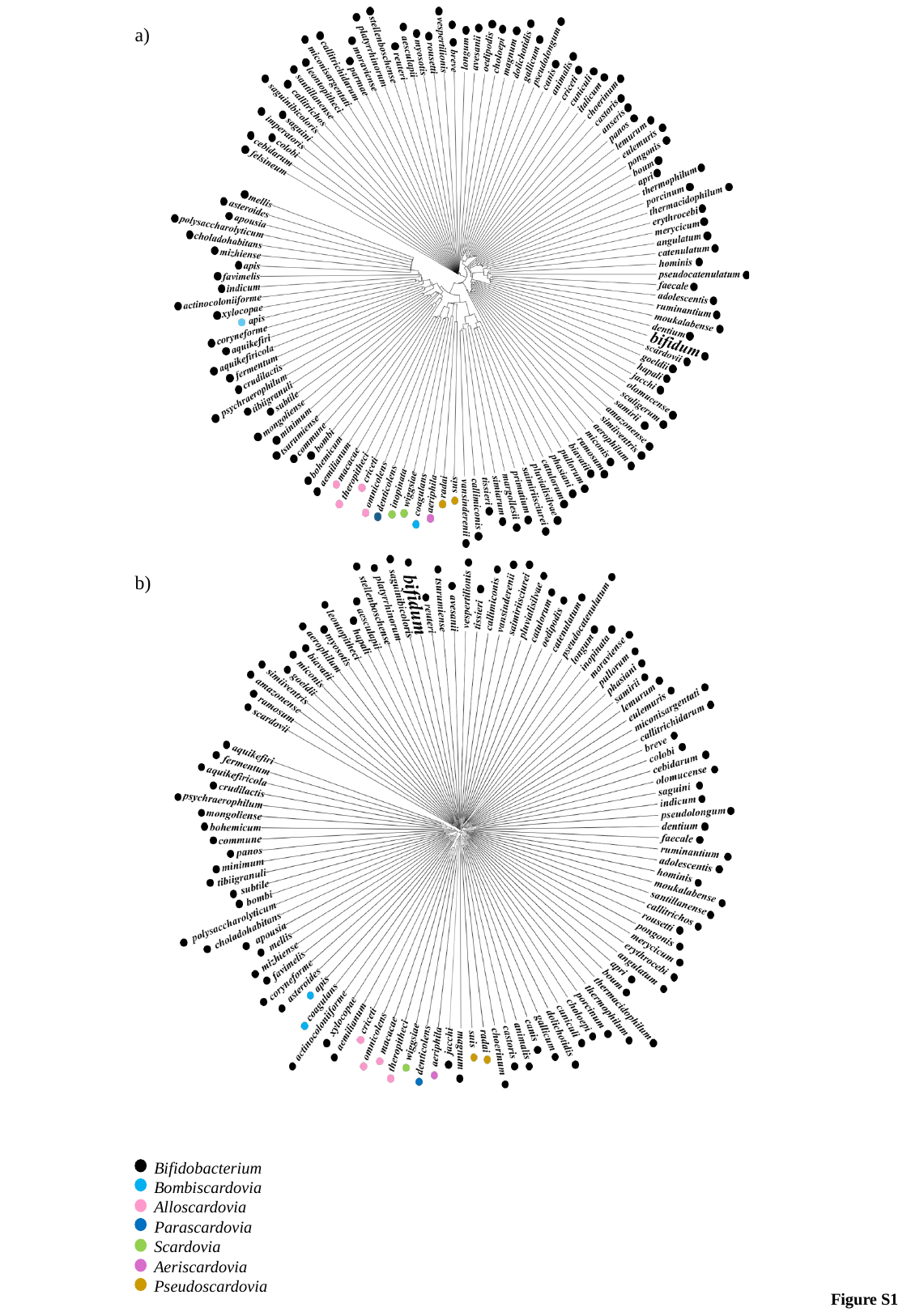

a)
b)
 Bifidobacterium
 Bombiscardovia
 Alloscardovia
 Parascardovia
 Scardovia
 Aeriscardovia
 Pseudoscardovia
Figure S1

## Slide 2
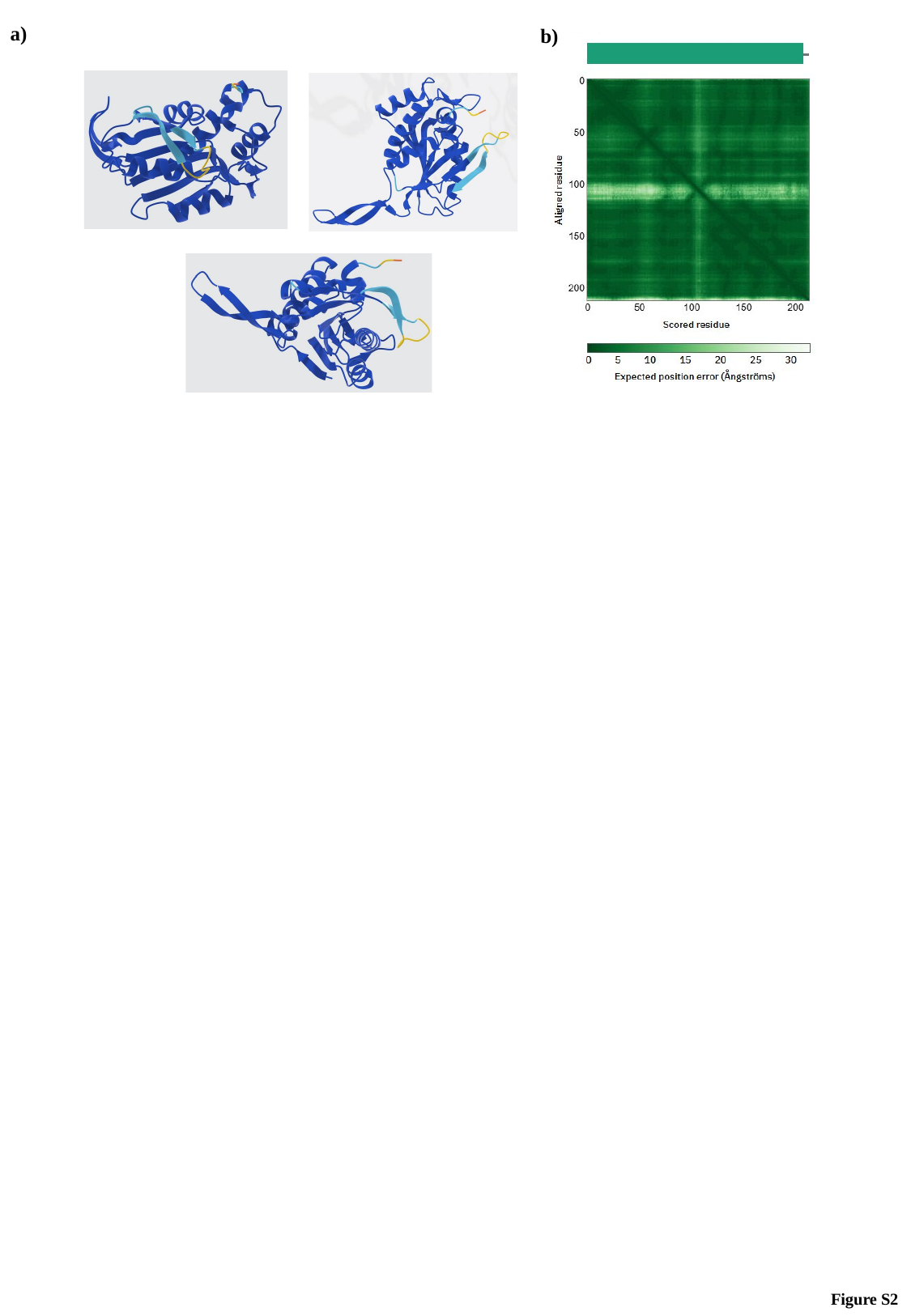

a)
b)
Figure S2
